# *ARNTL* hypermethylation promotes tumorigenesis and inhibits cisplatin sensitivity by activating *CDK5* transcription in nasopharyngeal carcinoma

**DOI:** 10.1186/s13046-018-0997-7

**Published:** 2019-01-08

**Authors:** Hao Peng, Jian Zhang, Pan-Pan Zhang, Lei Chen, Ling-Long Tang, Xiao-Jing Yang, Qing-Mei He, Xin Wen, Ying Sun, Na Liu, Ying-Qin Li, Jun Ma

**Affiliations:** 1 Department of Radiation Oncology, State Key Laboratory of Oncology in Southern China, Collaborative Innovation Center for Cancer Medicine, Guangdong Key Laboratory of Nasopharyngeal Carcinoma Diagnosis and Therapy, Sun Yat-sen University Cancer Center, Guangdong, 510060 People’s Republic of China; 2 Department of Experimental Research, State Key Laboratory of Oncology in Southern China, Collaborative Innovation Center for Cancer Medicine, Guangdong Key Laboratory of Nasopharyngeal Carcinoma Diagnosis and Therapy, Sun Yat-sen University Cancer Center, Guangdong, 510060 People’s Republic of China

**Keywords:** Nasopharyngeal carcinoma, *ARNTL*, Methylation, Proliferation, *CDK5*, Chemotherapy sensitivity

## Abstract

**Background:**

Increasing evidence support an important role for DNA methylation in nasopharyngeal carcinoma (NPC). Here, we explored the role of circadian clock gene *Aryl Hydrocarbon Receptor Nuclear Translocator-Like* (*ARNTL*) methylation in NPC.

**Methods:**

We employed bisulfite pyrosequencing to determine the epigenetic change of *ARNTL* in NPC cell lines and tissues. *ARNTL* mRNA and protein expression in cell lines and tissues were detected by real-time PCR and western blotting. Then, we constructed cell lines overexpressing *ARNTL* and knocked down *ARNTL* to explore its function and effect on chemotherapy sensitivity of NPC cell lines to cisplatin in vitro and vivo. Finally, we investigated the potential molecular mechanism of *ARNTL* by gene set enrichment analysis (GSEA), dual Luciferase reporter assay and chromatin immunoprecipitation assay.

**Results:**

*ARNTL* was hypermethylated, and its mRNA and protein were significantly down-regulated in NPC cell lines and tissues. When treated by 5-aza-2′-deoxycytidine, mRNA expression was up-regulated. Overexpression of *ARNTL* could suppress NPC cells proliferation in vitro and *vivo* while silencing of *ARNTL* using shRNA achieved opposite results. GSEA assay found that *ARNTL* was associated with cell cycle and ectopic *ARNTL* overexpression could induce G2-M phase arrest. Then, we identified and validated cyclin-dependent kinase 5 (*CDK5*) as the targeting gene of *ARNTL* by dual Luciferase reporter assay and chromatin immunoprecipitation assay. When transiently infected *ARNTL*-overexpression cells with PENTER-vector or PENTER-*CDK5* plasmids, the later could reverse the suppressive effects of *ARNTL* on NPC cell proliferation. Moreover, *ARNTL* significantly enhanced sensitivity to cisplatin in NPC cells.

**Conclusions:**

*ARNTL* suppresses NPC cell proliferation and enhances sensitivity to cisplatin by targeting *CDK5*. *ARNTL* may represent a novel therapeutic target for NPC.

**Electronic supplementary material:**

The online version of this article (10.1186/s13046-018-0997-7) contains supplementary material, which is available to authorized users.

## Background

Nasopharyngeal carcinoma (NPC) is a malignancy arising from the nasopharynx epithelium, and it is mainly endemic in Southeast Asia but relatively rare in white population [1]. Radiotherapy with or without chemotherapy has emerged as the standard care for non-disseminated disease. Although the advance in radiotherapy technique and chemotherapy strategies have improved prognosis of NPC to some extent, there are still approximately 30% of cases suffering treatment failure [2–4]. Identifying novel and effective treatments is essential for further improvement of survival outcomes. Given this, we should have a better understanding of the molecular mechanism underlying the pathogenesis and progression of NPC first.

There is a mass of evidence showing that DNA methylation, one kind of epigenetic alterations, plays an important role in cancer initiation and progression [5–7]. In NPC, dysregulated methylation of specific genes could facilitate tumor cell proliferation and metastasis [8, 9]. Since DNA methylation is a dynamic modification which could be formed or reversed by plenty of enzymes, it represents a promising biomarker for future diagnosis, prognosis prediction and therapeutic target in cancer [10, 11]. In our previous study, we identified that *Aryl Hydrocarbon Receptor Nuclear Translocator-Like* (*ARNTL*) is one of the top-ranked hypermethylated genes between 24 normal nasopharynx epithelium and 24 NPC tissues by Illumina Human Methylation 450 K Beadchips (GSE52068) [12]. *ARNTL*, also called *Bmal1*, is an indispensable core component of clock system [13], which forms a heterodimer with circadian genes and activates transcription of upstream genes [14, 15].

Circadian system consists of a series of genes including *Clock*, *Bmal1*, *Per1*, *Per2*, *Cry1*, *Cry2*, *Cry3* and *CK1e* [16]. In mammals, many physiological and behavioral processes display circadian rhythm which was controlled by endogenous clock system [17]. Disruption of circadian rhythm has been found to be associated with various human disease including cancer [18–20]. Among these genes, Previous studies found that abnormal expression of *ARNTL* was associated with tumor proliferation, cell cycle, survival outcomes as well as chemotherapy sensitivity in various cancers [16, 21–24], suggesting that *ARNTL* could act as a potential therapeutic target. However, the role of *ARNTL* in NPC remains unclear.

In this study, we provide our findings that *ARNTL* was downregulated in NPC cell lines and tumor tissues due to its promoter hypermethylation. Overexpression of *ARNTL* inhibited NPC cell proliferation by inducing G2/M phase arrest in vitro, and vice versa. Mechanism study revealed that *ARNTL* could suppress tumorigenicity through inhibiting cyclin-dependent kinase 5 (*CDK5*) transcription. Furthermore, *ARNTL* enhanced the sensitivity of NPC cells to cisplatin, suggesting that *ARNTL* may guiding the therapeutic timing of cisplatin in NPC.

## Methods

### Cell culture and clinical specimens

Human immortalized nasopharyngeal epithelial cell line NP69 was cultured in keratinocyte serum-free medium (Invitrogen, Life technologies, Grand Island, NY) supplemented with bovine pituitary extract (BD influx, Bioscience, USA). Human NPC cell lines (CNE1, CNE2, SUNE1, HONE1, HNE1, 5-8F, 6-10B) were maintained in RPMI-1640 (Invitrogen) supplemented with 5% fetal bovine serum (FBS, Gibco-BRL, Carlsbad, CA, USA). 293 T cells were grown in DMEM supplemented with 10% FBS. Additionally, 12 pairs of normal nasopharyngeal epithelial and freshly frozen NPC samples were obtained from our center. This study was authorized by the Institutional Ethical Review Boards of Sun Yat-sen University Cancer Center (YB2017–70), and written informed consents were provided by all patients for using their biopsy tissue samples.

### RNA extraction and reverse transcription quantitative PCR (RT-qPCR)

Total RNA was extracted from cultured cell lines using TRIzol Reagent (Invitrogen) according to the manufacturer’s instructions and reverse-transcribed to cDNA with M-MLV reverse transcriptase (Promega, Madison, WI, USA). Quantitative PCR reactions were performed using the Platinum SYBR Green qPCR SuperMix-UDG reagents (Invitrogen) and the CFX96 sequence detection system (Bio-Rad, Hercules, CA, USA) with the following primers: *ARNTL* forward, 5’-GATGGTTCAGTTTCATGAACC-3′ and reverse, 5’-CCTCTTATCCTGTGGATTTCC-3′; *CDK5* forward, 5’-CATCGTCAGGCTTCATGACG-3′ and reverse, 5’-CACCTCAGCTGAGTAACAGC-3′. GAPDH was applied as the endogenous control for normalization, and the 2^-△△CT^ was used to calculate the relative mRNA expression.

### Western blotting assay

Proteins were extracted from cells by using RIPA lysis buffer (Beyotime, Shanghai, China) and Bradford method was applied to test the concentration. A total of 20 μg proteins were separated by SDS-polyacrylamide gel electrophoresis (SDS-PAGE, Beyotime) and then transferred onto PVDF membranes (Millipore, Billerica, MA, USA). After transfer, the membrane was blocked in 5% defatted milk for 1 h and then incubated with primary anti-*ARNTL* (1,5000; Proteintech, Chicago, IL, USA) or anti-*CDK5* (1,1000; Abcam, Cambridge, UK) antibody overnight at 4 °C, followed by species-matched secondary antibodies for 1 h at room temperature. Finally, protein bands were visualized using enhanced chemiluminescence (Thermo, USA).

### DNA isolation and bisulfite pyrosequencing analysis

NPC cell lines (6 × 10^5^cells) were seeded on 100 mm culture dishes. After culturing for 24 h, the cells were treated with or without 10 μmol/L 5-aza-2′-deoxycytidine (DAC, Sigma Aldrich, Munich, Germany) for 72 h with replacing the drug and medium every 24 h. Subsequently, DNA was extracted using the EZ1 DNA Tissue Kit (Qiagen, Hilden, Germany) according to the manufacturer’s instructions. For bisulfite pyrosequencing analysis, 1–2 μg DNA was treated by sodium bisulfite using the EpiTect Bisulfite Kit (Qiagen). The PyroMark Assay Design Software 2.0 (Qiagen) was employed to design bisulfite pyrosequencing primers for *ARNTL* as follow: PCR forward: 5′-GGAAGGGGAGTGTTGGATAT-3′; PCR reverse: 5’-CCAAAACAACCCTAAATAACC-3′; sequencing primer: 5′- GGATATAGGAGTTTGTTGTTAA-3’.The PyroMark Q96 System (Qiagen) was adopted to conduct sequencing reaction and quantify methylation level.

### Construction of stable cell line and ARTNL short hairpin RNA (shRNA)

The pSin-EF2-puro-*ARNTL*, pSin-EF2-puro-vector and psin-EF2-puro-CDK5 plasmids were obtained from Land. Hua Gene Biosciences (Guangzhou, China), and the psin-EF2-puro-vector plasmid served as a control. To generate stable cell lines, lentivirus packing expression plasmids were co-transfected into 293 T cells for 48 h. Then, SUNE1 and HONE1 cell lines were infected with the supernatants containing virus for 48 h. After infection, stably infected cells were selected by puromycin (1 μg/ml) and further confirmed by RT-qPCR assay. Two shRNAs targeting *ARNTL* were purchased from Invitrogen and connected to pLKO.1 plasmid. The nucleotide sequences of shRNA#1 and shRNA#2 were shown in Additional file 1: Table S1.

### Cell proliferation and colony-formation assay

Cell proliferation assay was performed using CCK-8. Briefly, cells (1 × 10^3^) in 100 μl medium were seeded into 96-well plates and incubated for 0–4 days. Ten microliters CCK-8 (Dojindo, Tokyo, Japan) were added into each well, and the absorbance values were measured using a spectrophotometer at 450 nm. With regard to DDP treatment in vitro, 1 × 10^3^cells in 100 μl medium were incubated for 24 h first. Subsequently, the cells were treated with DDP at different concentrations (0, 0.625, 1.25, 2.5, 5 and 10 μg/ml) for 72 h according to our previous study [9], and the cell viability was determined. For the colony formation assay, 300 cells (600 cells for NP69) were seeded into 6-well plates with HONE1 cell line cultured for 1 week and SUNE1 cell line for 2 weeks. Then, the colonies were fixed in methanol and stained by crystal violet.

### Wound healing assay

SUNE-1 or HONE-1 cells were seeded into six-well plates for culture. When growing to almost confluent, the cells were subjected to serum-free medium starvation for 24 h. A sterile 200 μl tip was used to create artificial wounds by scraping the monolayers, and the cells were washed with PBS and then subjected to serum-free medium for another 24 h starvation. Images of cells migrating into wounds were captured at 0 h and 24 h by microscope (Olympus IX73, Japan).

### Transwell migration and invasion assays

Transwell chambers (8 μm pores, Corning, USA) coated with (invasion assay) or without (migration assay) Matrigel (BD Bioscience) were used to perform cell invasion and migration assays. SUNE-1 or HONE-1 cells (5 × 10^4^) in 200 μl serum-free medium were added into upper chambers, and the lower chambers were filled with 500 μl medium supplemented with 10% FBS. After incubating, the cells on upper membrane filter were fixed with methanol, stained with haematoxylin and counted.

### Cell cycle assay

For cell cycle analysis, 6 × 10^5^ cells were cultured onto 100 mm dishes for 24 h and then starved for 24 h by serum-free medium to synchronize cell cycle (G1/S). Thereafter, the cells were cultured using RPMI-1640 supplemented with 5% FBS for 9 h. Finally, cells were trypsinized, washed by cold PBS, fixed in 70% ethanol and stored at 4 °C for further analysis. A Cell Cycle Detection Kit (KeyGene BioTech, Nanjing, China) was used to conduct cell cycle analysis according to manufacturer’s instruction. Before staining, cells were washed twice by PBS, and thereafter RNase A and Propidium lodide (PI) mixture (1:9) was used to resuspend and incubate cells for 30–60 min at room temperature. The fluorescence intensity of the cells was determined using flow cytometry (Gallios; Beckman-Coulter, Germany).

### Gene set enrichment analysis (GSEA)

The GSEA (version 2.0.13, www.broadinstitute.org/gsea/) was applied to identity *ARNTL* expression related pathways in GSEA12452, and an enrichment score was calculated for each gene set (i.e., cell cycle pathway) by ranking each gene by their expression difference using computing a cumulative sum of each ranked in each gene set and recording the maximum deviation from zero as the enrichment score. We found that an overrepresentation of up- or downregulated genes between high and low *ARNTL* expression groups was associated with G2/M arrest pathway.

### Luciferase reporter assay

PGL3-based luciferase reporter plasmids (Promega) which contained wild type and mutant type of *CDK5* promoter were constructed. Thereafter, 2 × 10^5^ cells were seeded in 6-well plate for 24 h, and then each well were co-transfected with pGL3-basic (2 μg) and Renilla luciferase (20 ng) for 24 h using Lipofectamine 3000 (Invitrogen). The cells were harvested using Passive lysis buffer (Promega), and Dual Luciferase Reporter Assay System (Promega) was used to measure luciferase activity.

### Chromatin immunoprecipitation assay

Chromatin immunoprecipitation (ChIP) assay was performed in the SUNE1-ARNTL and HONE1 ARNTL cells using an EZ-Magna ChIP kit (Millipore) according to manufacturer’s protocol. Briefly, cells were induced crosslinking by 1% formaldehyde solution and then quenching crosslinking with 140 mM glycine. The nucleoprotein complexes were sheared to about 200–500 bp to yield DNA fragments and then immunoprecipitated with anti-ARNTL, IgG (negative control) or anti-RNA Pol II (positive control) antibodies overnight at 4 °C. PCR and real-time PCR assays were applied to detect the enrichment of DNA fragments in the binging sites of *CDK5* promoter. The primers used in PCR assay for detecting CDK5 promoter sequence were as follow: forward: 5’-TGCCCAACGAAATCACAAAGTCT-3′; reverse: 5’-CTTGAGGGCTATGGACCGAGGGA-3′. The percentage of binding DNA fragments was quantified related to input.

### Xenograft tumor models

Female BALB/c nude mice (4–5 weeks old) were purchased from Charles River Laboratories (Beijing, China) and bred at the Animal Experiment Center of Sun Yat-sen University. For tumor growth model, 1 × 10^6^ SUNE1-*ARNTL* or SUNE1-Vector cells were injected into the dorsal flank of mice (*n* = 7 in each group). Tumor size was measured every 3 days. Four weeks later, mice were sacrificed and tumors were weighted. For chemosensitivity assay in vivo, 1 × 10^6^ HONE1-*ARNTL* or HONE1-Vector cells were injected. After tumor formed in palpable mass 6 days after transplantation, the mice were randomly assigned to four groups (*n* = 5 in each group) which received intraperitoneal DDP (4 mg/kg) or normal saline injection every 3 days: ARNTL + DDP; ARNTL + saline; Vector + DDP; Vector + saline. Tumor size was also measured every 3 days. Two weeks later, mice were sacrificed and tumors were weighted. All tumor volumes were calculated as follow: volume = D × d^2^ × π/6, where D represents the longest diameter and d represents the shortest diameter. All animal assays were carried out in accordance with the detailed rules of the Animal Care and Use Ethics Committee of Sun Yat-sen University and we tried our best to minimize animal suffering.

### Immunohistochemistry assay

Formalin-fixed paraffin-embedded (FFPE) slices of xenograft mice tissues were used to perform Immunohistochemistry (IHC). Briefly, the tissues were deparaffinized at 60 °C for 30 min and then rehydrated. The endogenous peroxidase was blocked by 3% H_2_O_2_, and the tissues were treated by high-temperature citrate for antigen retrieval. Subsequently, non-specific binding was blocked and tissues were incubated with primary antibodies at 4 °C overnight. All slices were reviewed and scored by two experienced pathologists employed at our hospital.

### Statistical analysis

All data in our study were obtained from at least three independent experiments for each assay. Continuous variables were expressed as mean ± SD, and the difference was compared by non-parametric test. Spearman correlation analysis was used to analyze the relationship between *ARNTL* and *CDK5* mRNA expression in the GEO GSE12452 dataset and the TCGA dataset. The study data underlying current findings were deposited at the Research Data Deposit (RDDB2018000394, available at http://www.researchdata.org.cn). All tests were two-sided, and *P* < 0.05 was considered significant. Statistical analysis was conducted via SPSS 18.0 version (SPSS Inc., Chicago, IL, USA) software.

## Results

### *ARNTL* promoter is hypermethylated in NPC

**Fig. 1:**
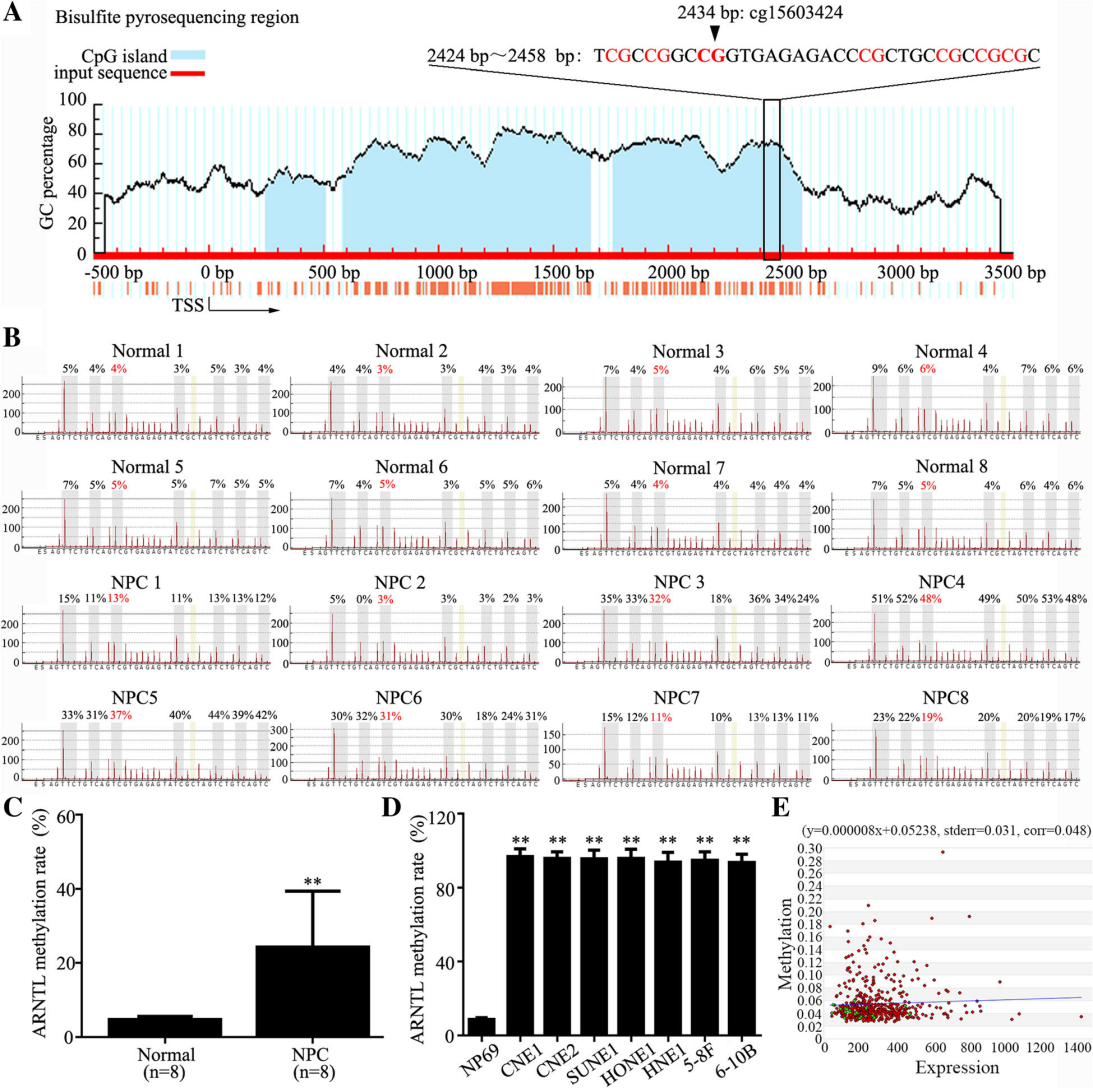
*ARNTL* is hypermethylated in nasopharyngeal carcinoma. **a** *ARNTL* promoter CpG islands and bisulfite pyrosequencing region. Blue region, CpG islands; Red region, input sequence; TSS, transcription start site; cg15603424, CG site identified in our previous genome-wide methylation analysis; red text, CG sites for bisulfite pyrosequencing; bold red text, the most methylated CG sites in *ARNTL*. **b** Bisulfite pyrosequencing analysis of the *ARNTL* promoter region in 8 pairs of normal and nasopharyngeal carcinoma tissues. **c** Average methylation levels of *ARNTL* in 8 pairs of normal and nasopharyngeal carcinoma tissues. **d** Methylation levels of *ARNTL* in NP69 and nasopharyngeal carcinoma cell lines. **e** Correlation between promoter methylation and mRNA expression of *ARNTL* in the TCGA head and neck cancer dataset. ** indicated *P* < 0.01

Based on our previous genome-wide methylation profile (12), we found that *ARNTL* CpG site (cg15603424) in its promoter region was obviously hypermethylated in NPC tissues compared to normal nasopharynx tissues, which was also found in the Hong-Kong dataset (Additional file 2: Figure S1). To validate this, bisulfite pyrosequencing analysis was used to detect the methylation level of *ARNTL* promoter in 8 pairs of NPC and normal nasopharynx tissues. The regions and CpG islands of *ARNTL* promoter region were presented in Fig. 1a. As shown in Fig. 1b and c, the methylation levels of *ARNTL* (cg15603424) in NPC were significantly higher than that in normal tissues (*P* < 0.001). Additionally, *ARNTL* promoter (cg15603424) methylation levels in NPC cell lines (CNE1, CNE2, SUNE1, HONE1, HNE1, 5-8F, 6-10B) were also significantly increased compared with that in human immortalized normal nasopharynx epithelial cell line (NP69) (Fig. 1d, Additional file 3: Figure S2; *P* < 0.001). These results identify that *ARNTL* promoter is hypermethylated in NPC. Further, the correlation between promoter methylation and mRNA expression of *ARNTL* in the TCGA head and neck cancer dataset was shown in Fig. 1e, which was opposite from that of our results. That should be attributed to the different biological characteristics between NPC and other head and neck cancers.

### *ARNTL* promoter hypermethylation results in its downregulation in NPC

**Fig. 2:**
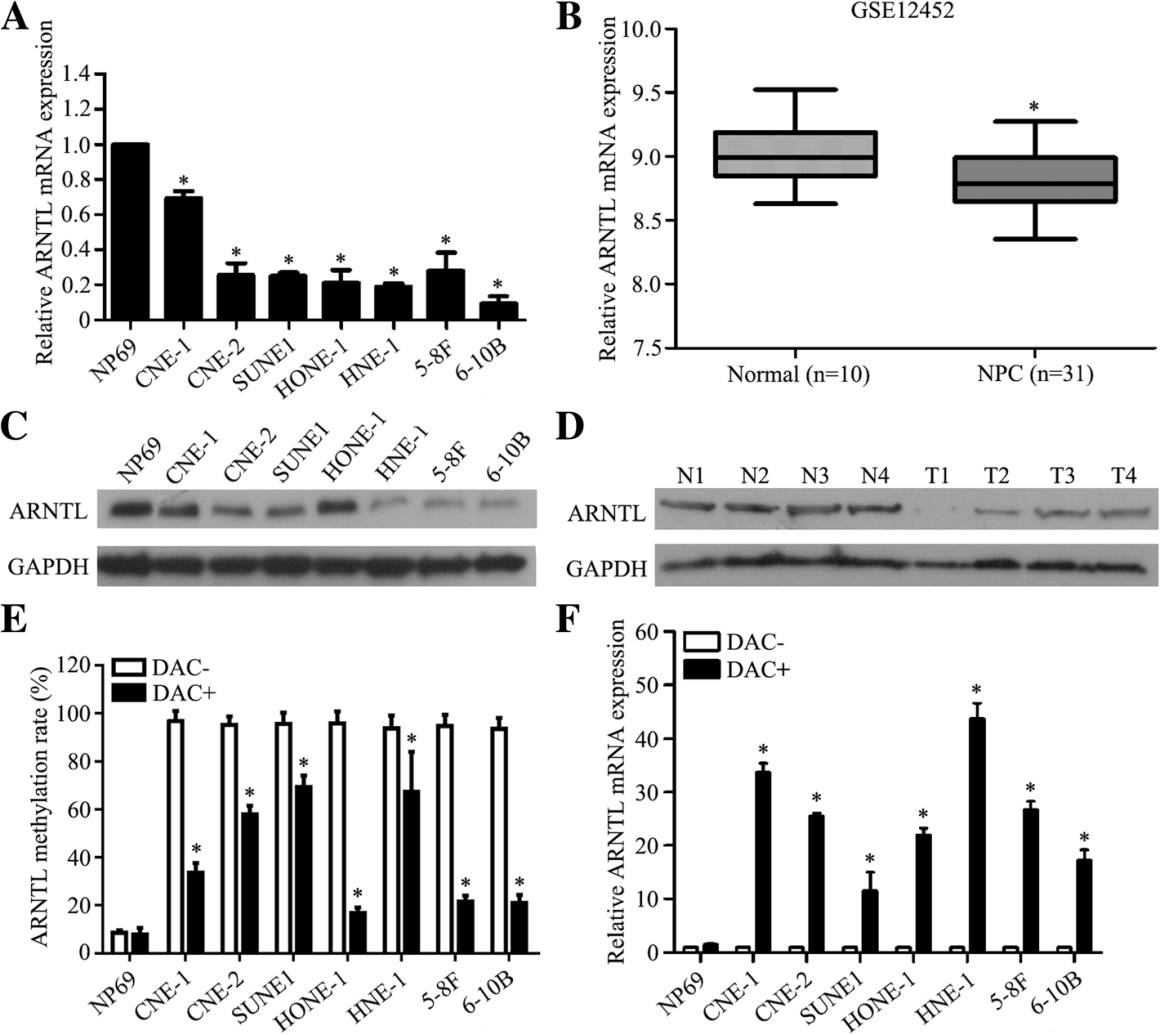
ARNTL is downregulated in nasopharyngeal carcinoma due to its promoter hypermethylation. **a** Quantitative RT-PCR analysis of *ARNTL* mRNA expression in NP69 and nasopharyngeal carcinoma cell lines. **b** *ARNTL* mRNA is downregulated in the GSE12452 nasopharyngeal carcinoma dataset. **c** Western blotting assay of *ARNTL* protein expression in nasopharyngeal carcinoma cell lines and normal NP69. **d** Western blotting assay of *ARNTL* protein expression in nasopharyngeal carcinoma tissues (T, *n* = 4) and normal nasophrynx epithelial tissues (T, *n* = 4). **e** *ARNTL* methylation levels before and after DAC treatment in NP69 and nasopharyngeal carcinoma cell lines. **f** *ARNTL* mRNA expression before and after DAC treatment in NP69 and nasopharyngeal carcinoma cell lines. * indicated *P* < 0.05

To know the expression of *ARNTL*, we firstly performed RT-qPCR to measure the mRNA expression and found that *ARNTL* mRNA was significantly downregulated in all NPC cell lines (Fig. 2a; *P* < 0.05). Results of analyzing external microarray-based high-throughput NPC datasets (GSE12452) further confirmed our findings that *ARNTL* was downregulated in NPC tissues (Fig. 2b; *P* < 0.05). Furthermore, results of western blotting revealed *ARNTL* protein expression in NPC cell lines and tissues was significantly downregulated than that in normal cell line and tissues (Fig. 2c, d; *P* < 0.05). To further characterize the association between ARNTL promoter methylation status and its expression, we used DAC to treat the cell lines. Compared with the NPC cell lines without DAC treatment, those treated by DAC had significantly lower *ARNTL* methylation (Fig. 2e, Additional file 4: Figure S3; *P* < 0.05); while the *ARNTL* mRNA expression was substantially increased (Fig. 2f; *P* < 0.05). Notably, these were no significant changes in normal NP69 cell line. Together, these results indicated that *ARNTL* promoter hypermethylation contributed to its downregulation.

### *ARNTL* suppresses NPC cell viability and colony formation in vitro

**Fig. 3:**
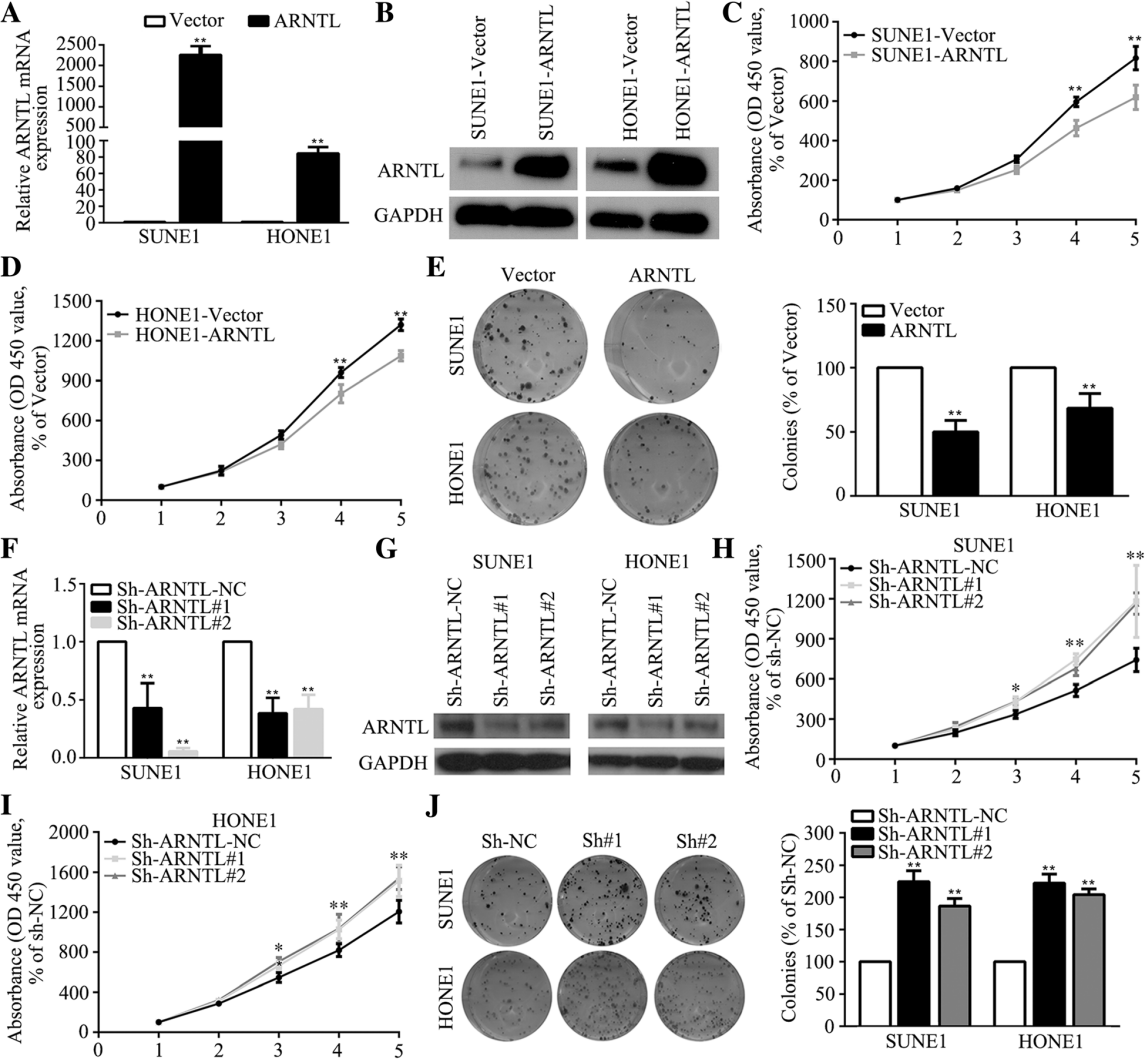
Effects of *ARNTL* overexpression or knocking down on nasopharyngeal carcinoma cell viability and colony formation ability in vitro. **a** Quantitative RT-PCR assay of *ARNTL* mRNA expression in SUNE1 and HONE1 cells stably overexpressing ARNTL. **b** Western blotting assay of *ARNTL* protein expression in SUNE1 and HONE1 cells stably overexpressing ARNTL. **c**, **d** The CCK-8 assay demonstrated that overexpression *ARNTL* reduced the viability of SUNE1 and HONE1 cells. **e** The colony formation assay showed that overexpression of *ARNTL* suppressed colony-forming ability of SUNE1 and HONE1 cells. **f** Quantitative RT-PCR analysis of *ARNTL* mRNA expression in SUNE1 and HONE1 cells knocking down by ShRNAs. **g** Western blotting analysis of *ARNTL* protein expression in SUNE1 and HONE1 cells knocking down by ShRNAs. **h**, **i** The CCK-8 assay demonstrated that knocking down *ARNTL* promoted the viability of SUNE1 and HONE1 cells. **j** The colony formation assay showed that knocking down *ARNTL* enhanced colony-forming ability of SUNE1 and HONE1 cells. * indicated *P* < 0.05; ** indicated *P* < 0.01

To evaluate the function of *ARNTL* in vitro, we constructed two cell lines (SUNE1, HONE1) which stably overexpressing *ARNTL* or Vector (Fig. 3a, b). The CCK-8 assay revealed that SUNE1 and HONE1 cells overexpressing *ARNTL* achieved significantly slower viability than that of cells overexpressing Vector (Fig. 3c, d). Moreover, overexpressing of *ARNTL* could remarkably reduce the colony formation abilities of SUNE1 and HONE1 cells (Fig. 3e). However, ARNTL had no or little influence on NPC cell invasion and migration (Additional file 5: Figure S4A-B). Then, we transiently transfected SUNE1 and HONE1 cells with shRNAs to downregulate *ARNTL* expression (Fig. 3f, g). The CCK-8 assay demonstrated that cells transfected with *ARNTL* shRNAs grew significantly faster than cells transfected with control shRNA (Fig. 3h, i). Similarly, knocking down *ARNTL* leads to stronger colony formation ability (Fig. 3j). These results suggested that *ARNTL* could inhibit NPC cell proliferation in vitro.

### *ARNTL* suppresses xenograft tumor growth in vivo

**Fig. 4:**
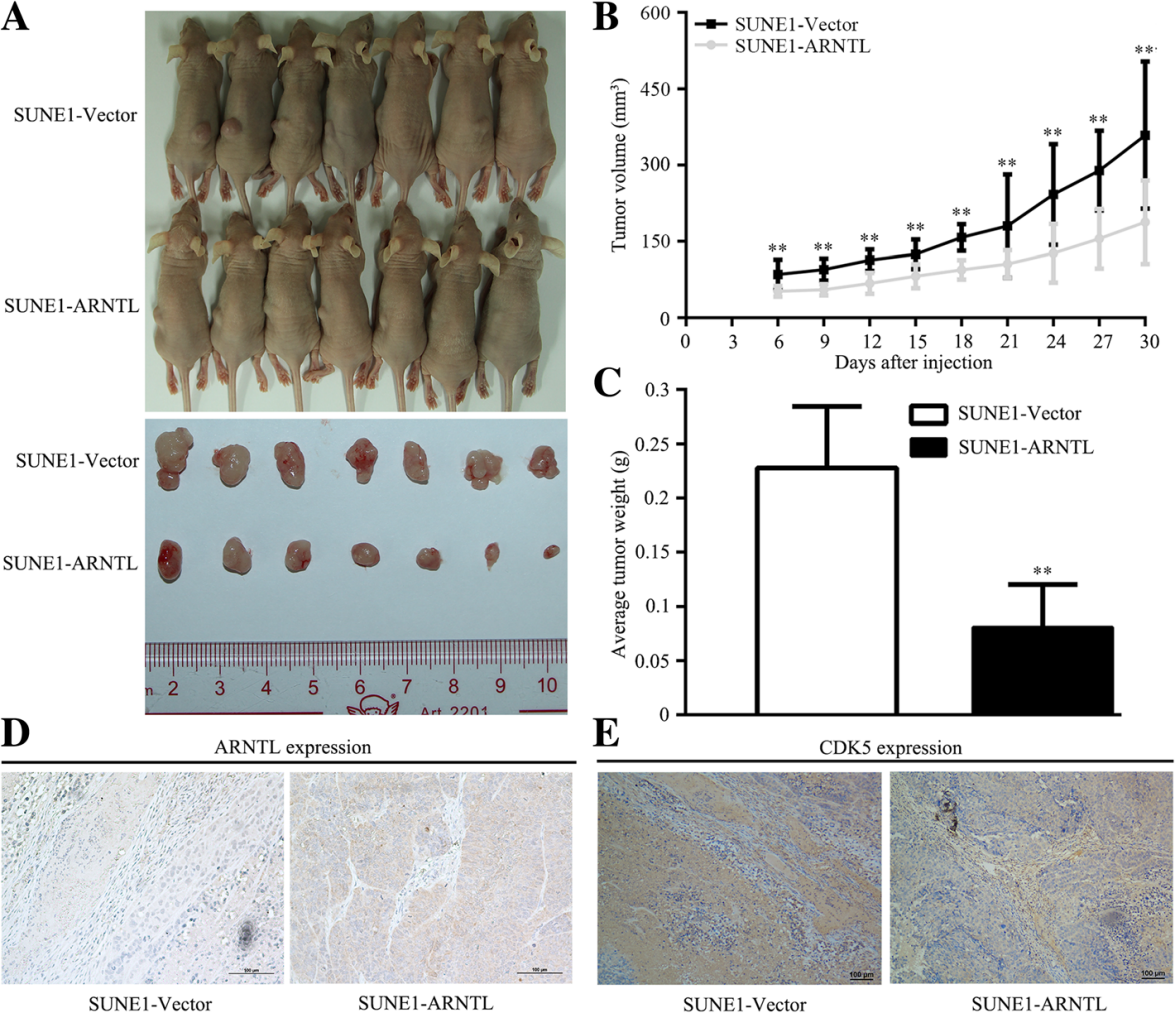
Overexpression of *ARNTL* suppressed tumorigenicity of nasopharyngeal carcinoma cells in vivo. **a** Xenograft tumors of BALB/c nude mice formed at 30 days after injecting with SUNE1 cells stably overexpressing *ARNTL* or Vector. **b** Growth curves of xenograft tumor volume. **c** Average xenograft tumor weights at 30 days after injecting with SUNE1 cells. **d** Immunohistochemistry assay of *ARNTL* protein expression in xenograft tumors. **e** Immunohistochemistry assay of *CDK5*protein expression in xenograft tumors. ** indicated *P* < 0.01

To further explore the role of *ARNTL* in vivo, we constructed a xenografted tumor growth model through injecting SUNE1 cells stably overexpressing *ARNTL* or vector into the dorsal flank of nude mice (*n* = 7 each group). The tumors in the mice injected with SUNE1 cells overexpressing *ARNTL* grew significantly slower and formed smaller volume than that in the mice injected with SUNE1 cells overexpressing Vector (Fig. 4a, b). When we sacrificed the mice after four weeks, the tumor weight in the *ARNTL*-overexpressing group was substantially lighter than that in Vector-overexpressing group (0.081 ± 0.042 g vs. 0.229 ± 0.074 g; *P* = 0.001; Fig. 4c). Furthermore, IHC assay showed that *ARNTL* protein expression was significantly upregulated in xenograft tumor which injected with *ARNTL*-overexpression cells compared with that injected with Vector-overexpression cells (Fig. 4d). Taken these results together, *ARNTL* could reduce the tumor growth ability of NPC cells in vivo.

### *ARNTL* suppresses NPC cell proliferation by inducing G2-M phase arrest

**Fig. 5:**
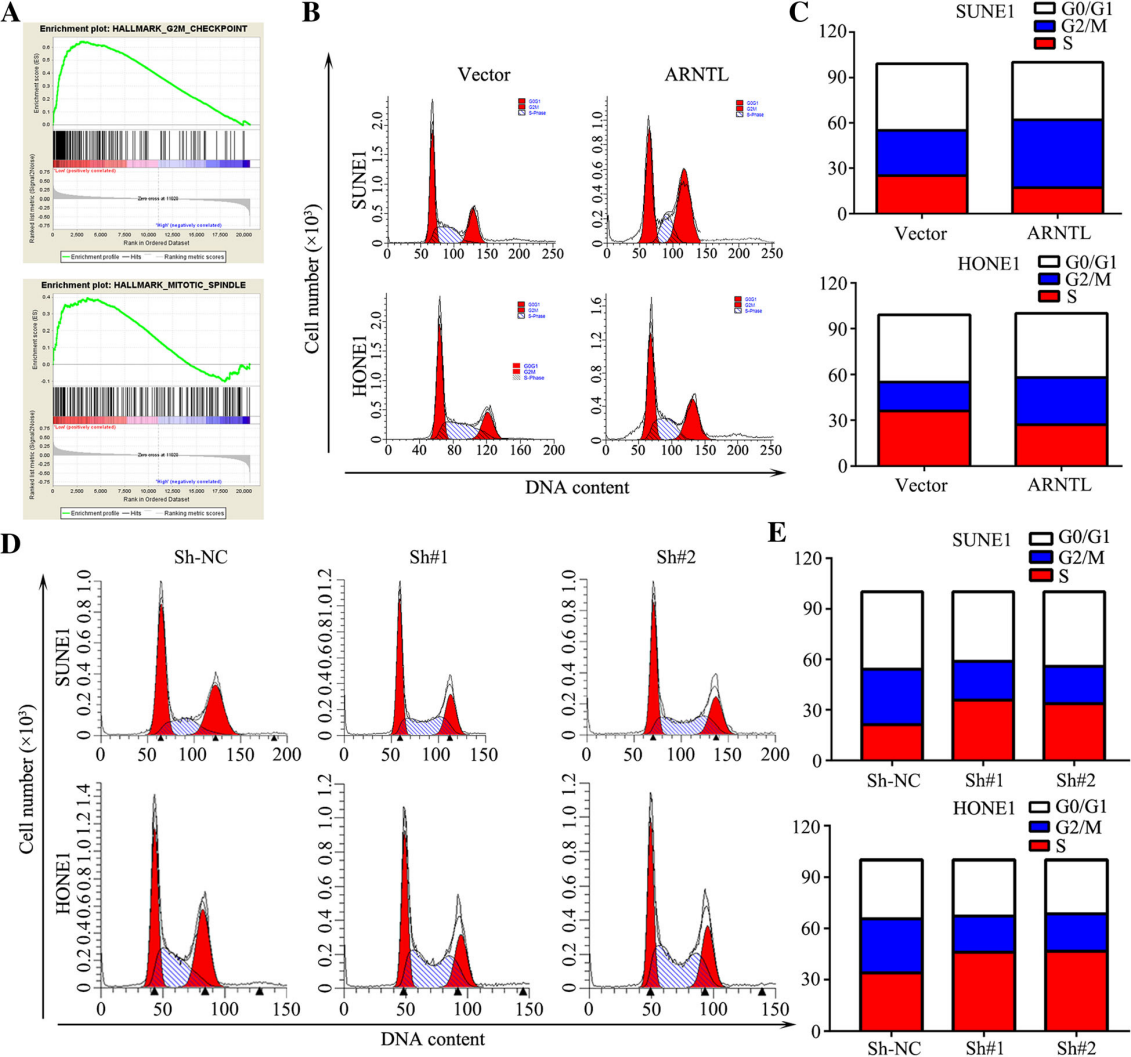
Overexpression of ARNTL induced cell cycle arrest at G2-M phase. **a** GSEA enrichment plots showed that enrichment of G2/M checkpoint and mitotic spindle pathways was associated with *ARNTL* downregulations. **b**, **c** Flow cytometry analysis of cell cycle distribution in SUNE1 and HONE1 cells stably overexpressing *ARNTL* or Vector. **d**, **e** Flow cytometry analysis of cell cycle distribution in SUNE1 and HONE1 cells after silencing *ARNTL*

To identify potential pathways which were responsible for *ARNTL* regulation on NPC cell proliferation, gene set enrichment analysis (GSEA) found that ARNTL expression was related to hallmarks of G2/M checkpoint and mitotic spindle genes in GSE12452 dataset (Fig. 5a). Actually, cancer-associated gene sets, including G2/M checkpoint and mitotic spindle pathways, were found to be enriched in *ARNTL*-high expression tumors but not in *ARNTL*-low expression tumors.

Flow cytometry assay showed that overexpression of *ARNTL* significantly increased the proportion of cells in G2/M phase (31.03% ± 0.91% vs. 19.12% ± 0.30% in HONE1 cells; 45.36% ± 0.55% vs. 30.09% ± 1.06% in SUNE1 cells; *P* < 0.05) and reduced the percentage of cells in S phase (27.15% ± 0.87% vs. 36.13% ± 0.27% in HONE1 cells; 17.04 ± 0.33% vs. 25.11% ± 0.53% in SUNE1 cells; *P* < 0.05) (Fig. 5b, c). However, knocking down *ARNTL* could decrease the proportion of cells in G2/M phase (31.63% ± 0.33% vs. 21.24% ± 1.09% vs. 21.93% ± 0.48% in HONE1 cells; 33.06% ± 0.45% vs. 23.14% ± 0.58% vs. 22.20% ± 0.90% in SUNE1 cells; *P* < 0.05) and increase the percentage in S phase (33.99% ± 0.14% vs. 46.0% ± 0.94% vs. 46.66% ± 0.67% in HONE1 cells; 21.12% ± 1.18% vs. 35.73% ± 0.37% vs. 36.99% ± 0.26% in SUNE1 cells; *P* < 0.05; Fig. 5d-e). Collectively, these results suggested ARNTL suppress cell proliferation by inducing G2/M phase arrest.

### *ARNTL* suppresses NPC cell proliferation by inhibiting *CDK5* transcription

**Fig. 6:**
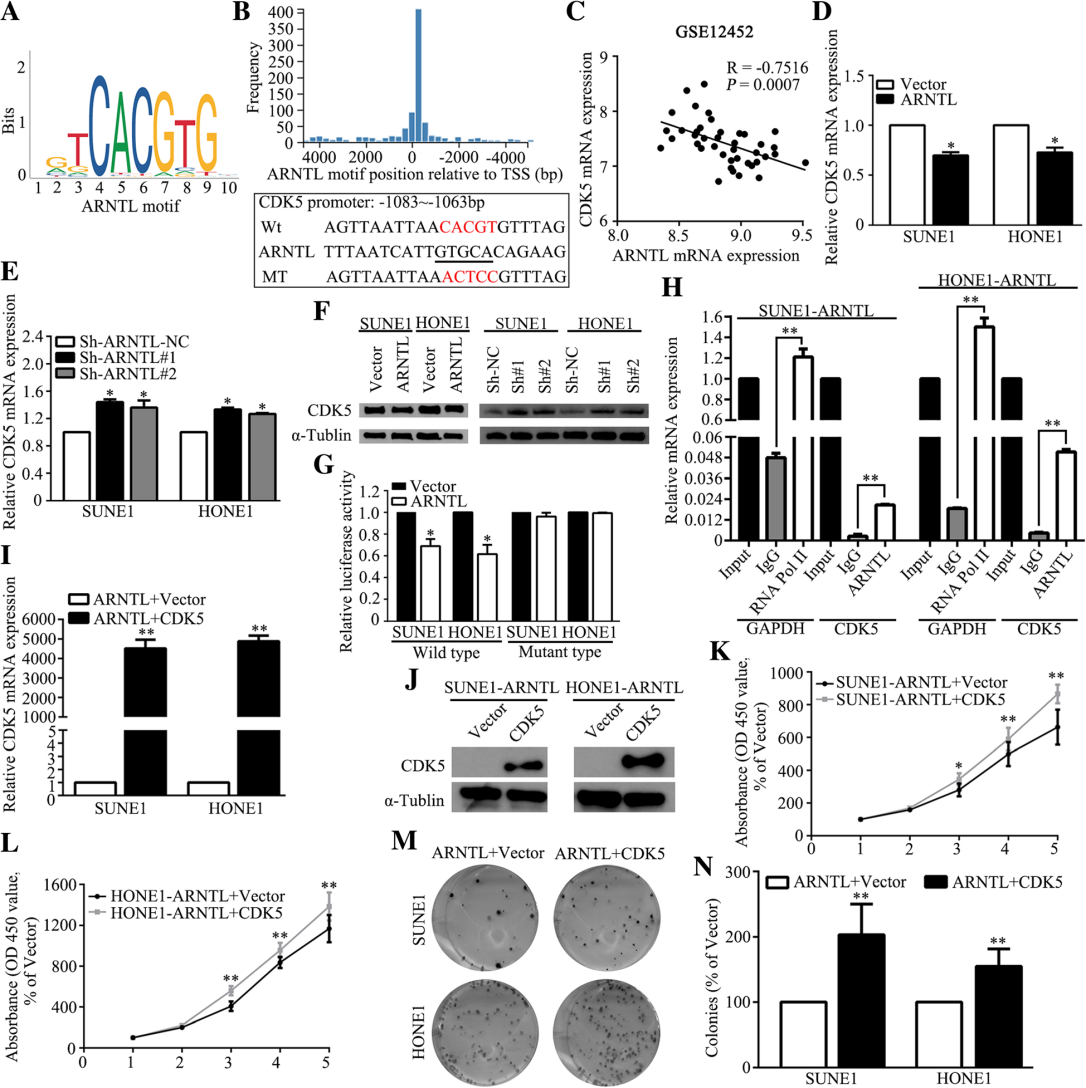
*CDK5* is a direct targeting gene of *ARNTL*. **a** *ARNTL* motif; **b** Putative *ARNTL*-binding sequence in promoter of *CDK5* mRNA; **c** Spearman correlation analysis between *ARNTL* and *CDK5* mRNA expression in GSE12452 nasopharyngeal carcinoma dataset; **d** Quantitative RT-PCR analysis of *CDK5* mRNA expression in *ARNTL*-overexpression SUNE1 and HONE1 cells; **e** Quantitative RT-PCR analysis of *CDK5* mRNA expression in *ARNTL*-silencing SUNE1 and HONE1 cells; **f** Western blotting analysis of *CDK5* protein expression in *ARNTL*-overexpression or *ARNTL*-silencing SUNE1 and HONE1 cells;. **g** Relative luciferase activity of *ARNTL*-overexpression or Vector-expression SUNE1 and HONE1 cells after transfecting with wild type or mutant *CDK5* 3’UTR reporter genes. **h** ChIP real-time PCR assay for assessing the enrichment of *ARNTL* in the CDK5 promoter regimen in SUNE1-*ARNTL* and HONE1-*ARNTL* NPC cells (anti-RNA Pol II serves as positive control).; **i** Quantitative RT-PCR analysis of *CDK5* mRNA expression in *ARNTL*-overexpression SUNE1 and HONE1 cells after transiently transfecting with *CDK5*-overexpression plasmid. **j** Western blotting analysis of *CDK5* protein expression in *ARNTL*-overexpression SUNE1 and HONE1 cells after transiently transfecting with *CDK5*-overexpression plasmid. **k** The CCK-8 assay revealed overexpression of *CDK5* could reverse *ARNTL*-mediated viability suppression in SUNE1 cells; **l** The CCK-8 assay revealed overexpression of *CDK5* could reverse *ARNTL*-mediated viability suppression in HONE1 cells; (**m-n**) Colony formation assay revealed that CDK5 could reverse *ARNTL*-mediated colony-forming ability suppression in SUNE1 and HONE1 cells. * indicated *P* < 0.05; ** indicated *P* < 0.01

To further analyze the underlying mechanism by which *ARNTL* suppress tumor proliferation, we employed the online JASPAR database (http://jaspar.genereg.net/cgi-bin/jaspar_db.pl) to identify potential downstream target genes. We found that *ARNTL* motif (Fig. 6a) could bind to the promoter of *CDK5* (Fig. 6b), a G2/M checkpoint gene which was reported to involve into G2/M cell cycle in many malignancies [25–27]. Therefore, we speculated that *CDK5* may act as the downstream target gene of *ARNTL*. Spearman correlation analysis of GSE12452 dataset found that *ARNTL* mRNA expression was negatively correlated with *CDK5* mRNA expression (Fig. 6c). Additionally, we also validated this negative correlation in many other cancers using The Cancer Genome Atlas (TCGA) dataset (Additional file 6: Figure S5). Moreover, overexpression of *ARNTL* could reduce the expression of *CDK5* while knocking down *ARNTL* could increase its expression in both mRNA (Fig. 6d, e) and protein levels (Fig. 6f). These results confirmed that *CDK5* expression was negatively correlated with *ARNTL* expression.

Then, we performed dual luciferase reporter gene assay to further validate our findings. The wild-type or mutant sequences of the *CDK5* promoter were cloned into luciferase reporter vectors. When transfected into *ARNTL*- or Vector-overexpression cells, the luciferase activity of wild-type reporter gene in ARNTL-overexpression cells was significantly lower than that in Vector-overexpression cells, while the luciferase activity of mutant reporter gene did not significantly differ between the two groups (Fig. 6g). Moreover, the ChIP assay also showed that *ARNTL* could bind to the promoter region of *CDK5* (Fig. 6h). Given these findings, *CDK5* was a potential target gene of *ARNTL*.

### *CDK5* reverses *ARNTL*-mediated proliferation suppression

To explore whether *CDK5* was a functional target in *ARNTL*-mediated tumor cell proliferation suppression, we transiently infected the SUNE1 or HONE1 *ARNTL*-overexpression cells with PENTER-vector or PENTER-*CDK5* plasmids (Fig. 6i, j). The CCK-8 (Fig. 6k, l) and colony formation (Fig. 6m, n) assays demonstrated that the cells infected with *CDK5* achieved significantly stronger viability and colony formation ability compared with the cells transfected with Vector. Furthermore, we evaluated whether *ARNTL* could affect cell proliferation ability in *CDK5* downregulated cells. Western blot assay confirmed that *CDK5* was downregulated in NP69 cells compared with SUNE1 and HONE1 cell lines (Additional file 7: Figure S6A). Therefore, we knocked down *ARNTL* expression in NP69 cell (Additional file 7: Figure S6B). The CCK-8 (Additional file 7: Figure S6C) and colony formation (Additional file 7: Figures. S6D-E) assays showed that NP69 cells transfected with *ARNTL* ShRNAs achieved significantly stronger viability and colony formation ability compared with the cells transfected with control ShRNA. These results revealed that *CDK5* serves as a functional target of *ARNTL* and ectopic expression of *CDK5* could partially reverse the suppressive effect of *ARNTL*.

### *ARNTL* enhances sensitivity of NPC cells to cisplatin

**Fig. 7:**
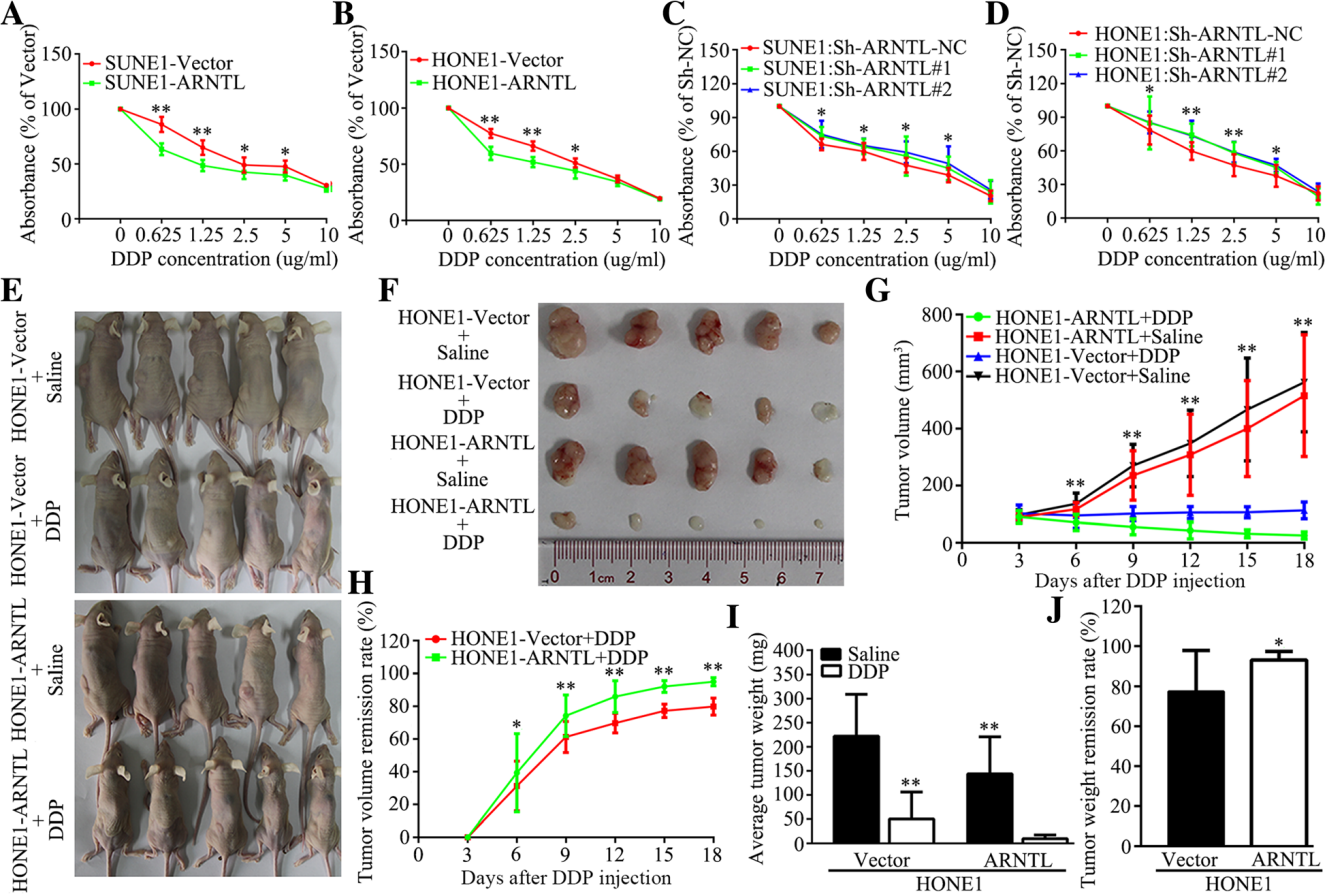
*ARNTL* increased sensitivity of nasopharyngeal carcinoma cells to cisplatin in vitro and vivo. **a**-**d** The CCK-8 assay revealed that overexpression of *ARNTL* increased cisplatin sensitivity while knocking down *ARNTL* decreased cisplatin sensitivity of SUNE1 and HONE1 cells in vitro. **e**, **f** Xenograft tumors of BALB/c nude mice at 18 days after injecting with Vector-overexpression or *ARNTL*-overexpression HONE1 cells and treating with cisplatin or saline. **g** Average tumor volume of BABL/c nude mice injecting with Vector-overexpression or *ARNTL*-overexpression HONE1 cells and treating with cisplatin or saline. **h** Tumor volume remission rates between Vector-overexpression and *ARNTL*-overexpression nude mice treated by cisplatin. **i** Average xenograft tumor weight of Vector-overexpression and *ARNTL*-overexpression nude mice treated by cisplatin or saline. **j** Tumor weight remission rates between Vector-overexpression and *ARNTL*-overexpression nude mice treated by cisplatin. * indicated *P* < 0.05; ** indicated *P* < 0.01

As previous studies found that *ARNTL* had an effect on chemosensitivity [24, 28], we therefore determined whether *ARNTL* could enhance the sensitivity of NPC cells to cisplatin. The CCK-8 assay showed that overexpression of *ARNTL* significantly enhanced the sensitivity while silencing of *ARNTL* substantially reduced the sensitivity of NPC cells to cisplatin in vitro (Fig. 7a-d). This function was further explored in a xenograft tumor model which was treated by cisplatin or normal saline. Compared with the Vector-overexpression group treated by cisplatin, the *ARNTL*-overexpression group treated by cisplatin achieved significantly greater tumor volume and weight remission in vivo (Fig. 7e-j). Taken these together, *ARNTL* could increase the sensitivity of NPC cells to cisplatin.

## Discussion

In our current study, we validate that *ARNTL* is downregulated in both NPC cell lines and freshly frozen tissues as a result of its promoter hypermethylation. Overexpression of *ARNTL* could reduce NPC cell viability and colony formation while silencing of *ARNTL* produces inverse effect in vitro. Xenograft tumor model also shows that *ARNTL* suppress NPC cell proliferation in vivo. Then, we identify that ARNTL induces G2/M phase arrest by targeting *CDK5*. Moreover, ARNTL could enhance the sensitivity of NPC cells to cisplatin in both *vitro* and *vivo*. Our findings provide new insights into potential mechanism of *ARNTL* regulating cell proliferation and clinical treatment for NPC.

Distant metastasis are the main failure patterns in NPC. Although the application of intensity-modulated radiotherapy has improved the management of NPC, there are still almost 30% of patients experiencing treatment failure [3, 4, 29, 30]. Therefore, better understanding of the molecular mechanisms underlying distant metastasis could facilitate future treatments for NPC. There is increasing evidence supporting that epigenetic processes play a vital role in cell biology and tissue physiology. With regard to cancer in human, these epigenetic processes are mainly exemplified by aberrant DNA methylation [11, 31]. Also, aberrant epigenetic changes play an important role in NPC [32]. Recently, numerous studies have uncovered the diagnostic and prognostic value of hypermethylation of some genes in NPC [8, 9, 12, 33]. Given these knowledge, it’s worth exploring the mechanism of specific gene methylation which contributes to disease progression or recurrence in NPC.

*ARNTL*, a member of the bHLH-PAS structural domain transcription factor family, mainly regulates cell circadian rhythm. Promoter hypermethylation of *ARNTL* was observed in breast cancer and hematologic malignancies [16, 34]. Consistent with these findings, our study also validated that *ARNTL* was hypermethylated in NPC. We found that *ARNTL* mRNA and protein expression were significantly downregulated in NPC cell lines and tissues. Overexpression of *ARNTL* could suppress NPC cells proliferation both in vitro and *vivo* while silencing of *ARNTL* substantially promoted NPC cells viability and colony formation. These findings suggest that *ARNTL* serve as a tumor suppressor in NPC, which is consistent with its role in other malignancies [21, 22, 24, 35].

Normal physiological cell cycle is mainly controlled by the protein kinase complexes consisting of cyclins and cyclin-dependent kinases (CDKs). Cyclins act as the regulatory subunit and CDKs serve as the catalytic subunit to activate the heterodimer complexes, which regulate entry into the S phase of cell cycle [36]. Aberrant expression of cell cycle components could lead to uncontrolled cell cycle, therefore resulting in uncontrolled cell proliferation and cancer [37]. Recent studies demonstrate that *ARNTL* is correlated with cell cycle [16, 22, 24, 38, 39]. Mullenders et al. and Grechez-Cassiau et al. revealed *ARNTL* affect the cell cycle and proliferation through p53/p21 signaling pathway [22, 39]. Zeng et al. found that *ARNTL* could regulate G2-M phase arrest by activating the ATM signaling pathway [24]. Our study also validated that *ARNTL* could induce G2-M phase arrest, further validating its role in cell cycle.

*CDK5*, a member of CDK family which binds to ATP sandwiched between N- and C-terminal lobes [40] and is activated by binding to non-cyclin *CDK5* activators CDK5R1 (p35) and CDK5R2 (p39) [41]. Recent evidence found that *CDK5*-induced G2/M arrest played an important role in cancer progression [42–45]. In our study, GSEA and flow cytometer found overexpression of *ARNTL* could induce G2-M phase arrest. To further clarify the underlying downstream *ARNTL,* luciferase reporter system and function rescue experiments validated *ARNTL* could inhibit NPC cell proliferation by directly regulating *CDK5* transcription. Combining these studies with our findings, it’s reasonable to infer that *ARNTL* induces G2-M phase arrest by binding to *CDK5* to inhibit NPC cell proliferation.

Chemotherapy is one of the most important treatments for malignancies, and resistance to chemotherapy could result in tumor recurrence or distant metastasis. Therefore, identifying potential target genes which correlate with chemotherapy sensitivity is of great importance to cancer care. Cisplatin has been the most widely and effective chemotherapy reagent for NPC for the past decades. Therefore, we explored whether *ARNTL* affected chemotherapy sensitivity of NPC cell to cisplatin. The results suggested that *ARNTL* could increase sensitivity to cisplatin both in vitro and *vivo*, consistent with previous findings that *ARNTL*-overexpression tumors showed improved sensitivity to anticancer drugs [24, 28, 46]. Our findings suggested that *ARNTL* could serve as a molecular target to increase cisplatin sensitivity in NPC.

## Conclusion

In summary, our study demonstrates that *ARNTL* could suppress NPC cell proliferation by targeting *CDK5* and inducing G2-M phase arrest. Moreover, ARNTL could enhance chemotherapy sensitivity of NPC cell to cisplatin. Our findings provide new insights into molecular mechanism of NPC progression and identify *ARNTL*/*CDK5* pathway as a novel target for treatment.

## Additional files

Additional file 1: **Table S1.** Nucleotide sequence of the ShRNA#1 and ShRNA#2 targeting ARNTL. (DOCX 13 kb)

Additional file 2: **Figure S1.** *ARNTL* methylation levels in the GSE52068 and GSE62366 nasopharyngeal carcinoma datasets. (TIF 109 kb)

Additional file 3: **Figure S2.** Bisulfite pyrosequencing analysis of the *ARNTL* promoter methylation in NP69 and nasopharyngeal carcinoma cell lines before DAC treatment. (TIF 396 kb)

Additional file 4: **Figure S3.** Bisulfite pyrosequencing analysis of the *ARNTL* promoter methylation in NP69 and nasopharyngeal carcinoma cell lines after DAC treatment. (TIF 388 kb)

Additional file 5: **Figure S4.** Overexpression of *ARNTL* had no impact on nasopharyngeal carcinoma cells invasion and migration. (**A**) Images of Transwell invasion (left) and migration (right) assay with *ARNTL*-overexpression or Vector-overexpression SUNE1 and HONE1 cells. (**B**) Images of wound healing assay with *ARNTL*-overexpression or Vector-overexpression SUNE1 and HONE1 cells. (TIF 6218 kb)

Additional file 6: **Figure S5.** Correlation between ARNTL expression and CDK5 expression in other cancers using The Cancer Genome Atlas (TCGA) dataset analysis. (TIF 1391 kb)

Additional file 7: **Figure S6.** *ARNTL* affect the proliferation ability of NP69 cells. (**A**) Western blot assay found that *CDK5* is downregulated in NP69 cells; (**B**) Knocking down *ARNTL* expression in NP69 cells; (**C**) CCK-8 assay demonstrated that knocking down *ARNTL* could promote NP69 cells proliferation; (**D-E**) Colony formation assay showed that knocking down *ARNTL* could promote NP69 cells proliferation. (TIF 3612 kb)

## Abbreviations

*ARNTL*: Aryl Hydrocarbon Receptor Nuclear Translocator-Like
CDK5: Cyclin-dependent kinase 5
DAC: 5-aza-2′-deoxycytidine
FBS: Fetal bovine serum
FFPE: Formalin-fixed paraffin-embedded
GSEA: Gene set enrichment analysis
IHC: Immunohistochemistry
NPC: Nasopharyngeal carcinoma
PI: Propidium lodide
SDS-PAGE: SDS-polyacrylamide gel electrophoresis
Sh-RNA: Short hairpin RNA
