# Correction to: *ARNTL* hypermethylation promotes tumorigenesis and inhibits cisplatin sensitivity by activating *CDK5* transcription in nasopharyngeal carcinoma

**DOI:** 10.1186/s13046-021-02238-5

**Published:** 2022-01-21

**Authors:** Hao Peng, Jian Zhang, Pan-Pan Zhang, Lei Chen, Ling-Long Tang, Xiao-Jing Yang, Qing-Mei He, Xin Wen, Ying Sun, Na Liu, Ying-Qin Li, Jun Ma

**Affiliations:** 1 Department of Radiation Oncology, State Key Laboratory of Oncology in Southern China, Collaborative Innovation Center for Cancer Medicine, Guangdong Key Laboratory of Nasopharyngeal Carcinoma Diagnosis and Therapy, Sun Yat-sen University Cancer Center, Guangdong, 510060 People’s Republic of China; 2 Department of Experimental Research, State Key Laboratory of Oncology in Southern China, Collaborative Innovation Center for Cancer Medicine, Guangdong Key Laboratory of Nasopharyngeal Carcinoma Diagnosis and Therapy, Sun Yat-sen University Cancer Center, Guangdong, 510060 People’s Republic of China

**Correction to: J Exp Clin Cancer Res 38, 11 (2019)**

**https://doi.org/10.1186/s13046-018-0997-7**

Following publication of the original article [1], the authors identified some minor errors in Supplemental Figs. 1 and 4, specifically:

- Fig. S1b: incorrect sample size listed; correct sample size is 25
- Fig. S4b: incorrect image used for SUNE1-ARNTL (24 h); correct image is now used

The authors provided the journal with their original data. The corrected figures are given here. The corrections do not have any effect on the final conclusions of the paper. The original article has been corrected.

## Supplementary Information

**Additional file 2: Fig. S1.** *ARNTL* methylation levels in the GSE52068 and GSE62366 nasopharyngeal carcinoma datasets.

**Additional file 5: Fig. S4.** Overexpression of *ARNTL* had no impact on nasopharyngeal carcinoma cells invasion and migration. (**A**) Images of Transwell invasion (left) and migration (right) assay with *ARNTL*-overexpression or Vector-overexpression SUNE1 and HONE1 cells. (**B**) Images of wound healing assay with *ARNTL*-overexpression or Vector-overexpression SUNE1 and HONE1 cells.
