# Supplementary material for: ARNTL hypermethylation promotes tumorigenesis and inhibits cisplatin sensitivity by activating CDK5 transcription in nasopharyngeal carcinoma
Source: J Exp Clin Cancer Res. 2019 Jan 8;38:11. doi: 10.1186/s13046-018-0997-7 (PMC6325889; doi:10.1186/s13046-018-0997-7)
Supplement: Supplementary file 1 — Table S1. Nucleotide sequence of the ShRNA#1 and ShRNA#2 targeting ARNTL. (DOCX 13 kb) [file 13046_2018_997_MOESM1_ESM.docx]

**Table S1**. Nucleotide sequence of the ShRNA#1 and ShRNA#2 targeting ARNTL.

| ShRNA | Nucleotide sequence |
| --- | --- |
| ShRNA#1 forward | CCGGGCTCCACTGACTACCAAGAAACTCGAGTTTCTTGGTAGTCAGTGGAGCTTTTTG |
| ShRNA#1 reverse | AATTCAAAAAGCTCCACTGACTACCAAGAAACTCGAGTTTCTTGGTAGTCAGTGGAGC |
| ShRNA#2 forward | CCGGGCAGAATGTCATAGGCAAGTTCTCGAGAACTTGCCTATGACATTCTGCTTTTTG |
| ShRNA#2 reverse | AATTCAAAAAGCAGAATGTCATAGGCAAGTTCTCGAGAACTTGCCTATGACATTCTGC |
