# Supplementary figures and images for: ARNTL hypermethylation promotes tumorigenesis and inhibits cisplatin sensitivity by activating CDK5 transcription in nasopharyngeal carcinoma
Source: J Exp Clin Cancer Res. 2019 Jan 8;38:11. doi: 10.1186/s13046-018-0997-7 (PMC6325889; doi:10.1186/s13046-018-0997-7)

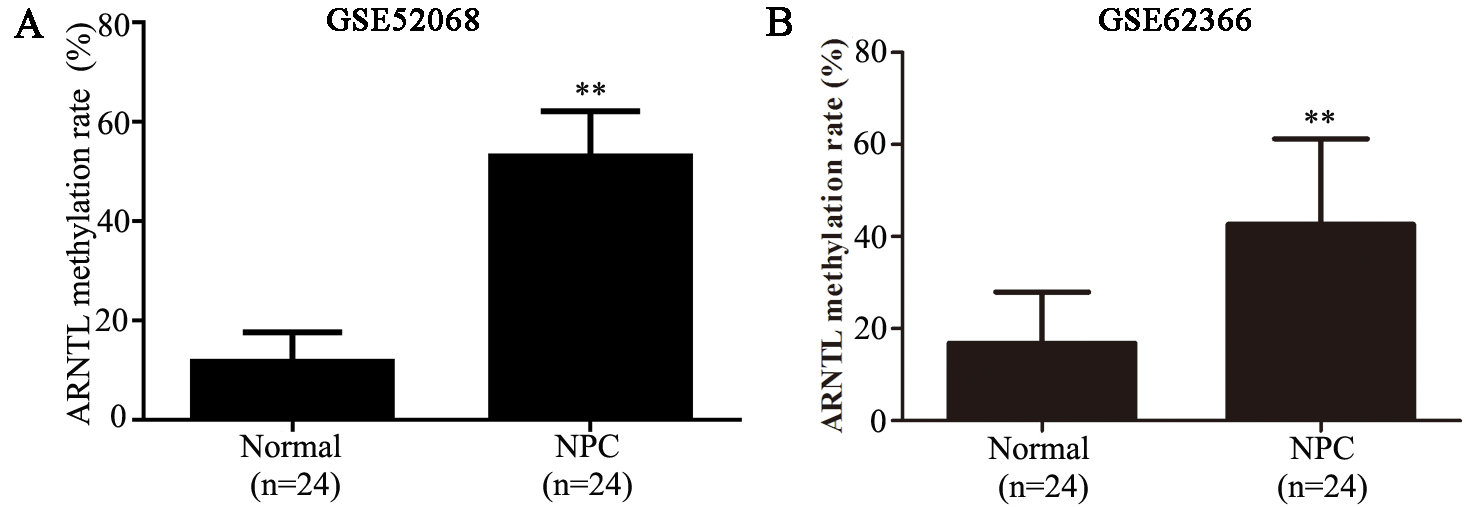

Supplement: Supplementary file 2 — Figure S1. ARNTL methylation levels in the GSE52068 and GSE62366 nasopharyngeal carcinoma datasets. (TIF 109 kb) [file 13046_2018_997_MOESM2_ESM.tif]

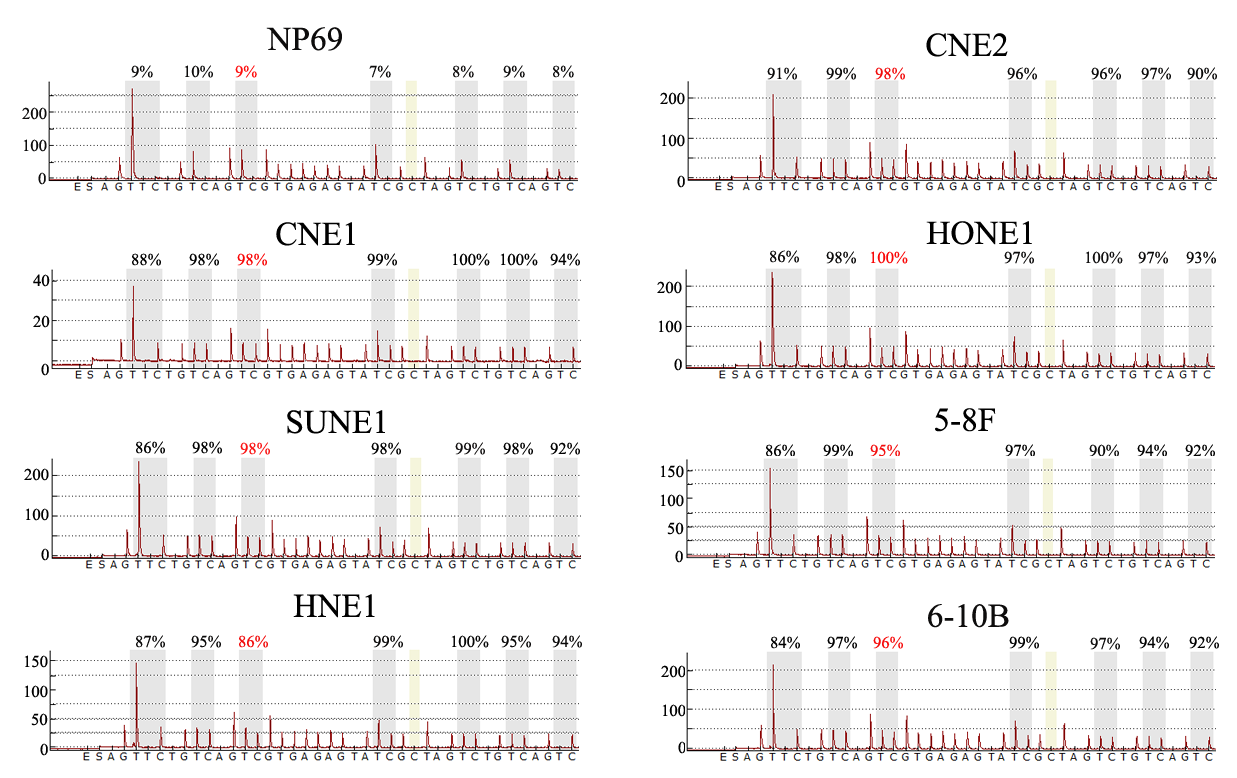

Supplement: Supplementary file 3 — Figure S2. Bisulfite pyrosequencing analysis of the ARNTL promoter methylation in NP69 and nasopharyngeal carcinoma cell lines before DAC treatment. (TIF 396 kb) [file 13046_2018_997_MOESM3_ESM.tif]

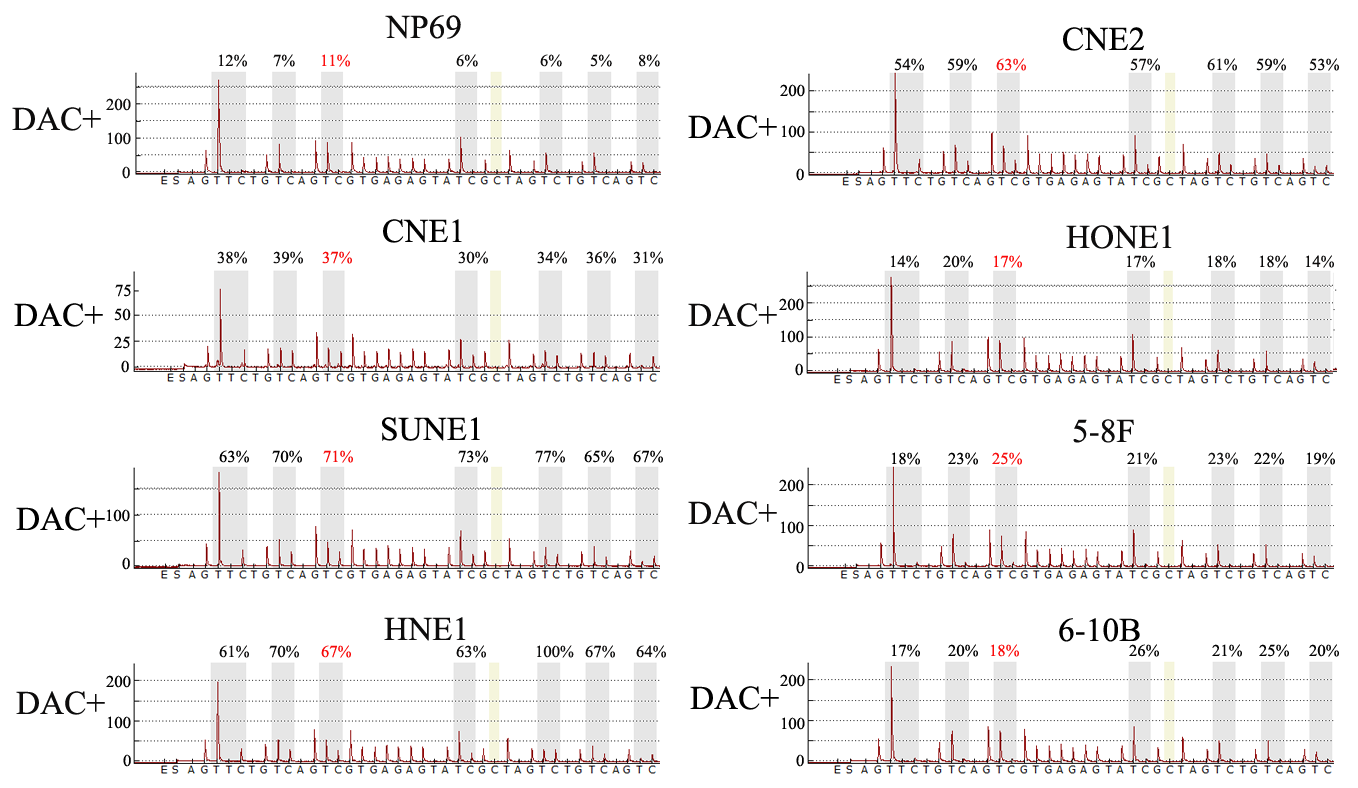

Supplement: Supplementary file 4 — Figure S3. Bisulfite pyrosequencing analysis of the ARNTL promoter methylation in NP69 and nasopharyngeal carcinoma cell lines after DAC treatment. (TIF 388 kb) [file 13046_2018_997_MOESM4_ESM.tif]

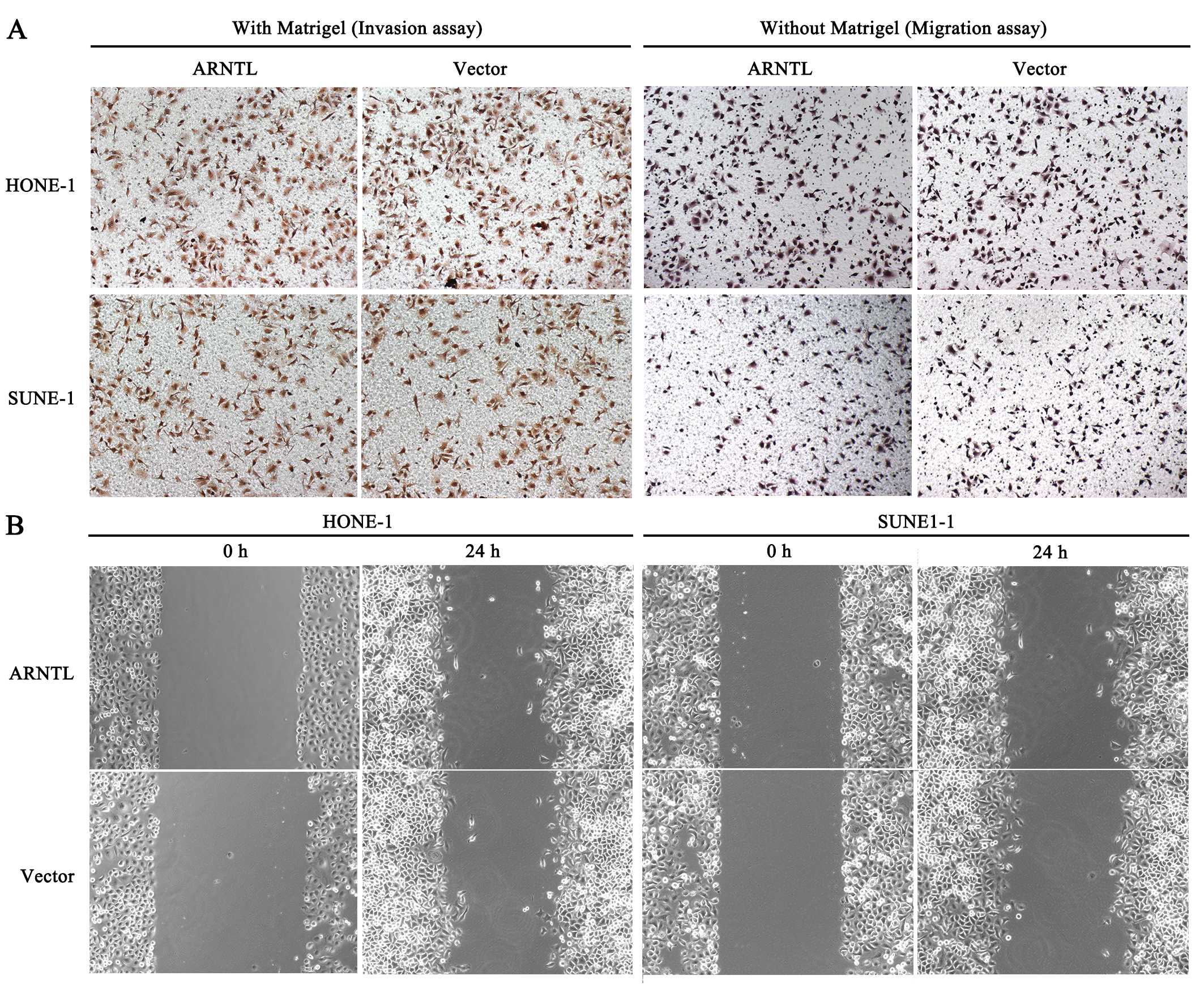

Supplement: Supplementary file 5 — Figure S4. Overexpression of ARNTL had no impact on nasopharyngeal carcinoma cells invasion and migration. (A) Images of Transwell invasion (left) and migration (right) assay with ARNTL-overexpression or Vector-overexpression SUNE1 and HONE1 cells. (B) Images of wound healing assay with ARNTL-overexpression or Vector-overexpression SUNE1 and HONE1 cells. (TIF 6218 kb) [file 13046_2018_997_MOESM5_ESM.tif]

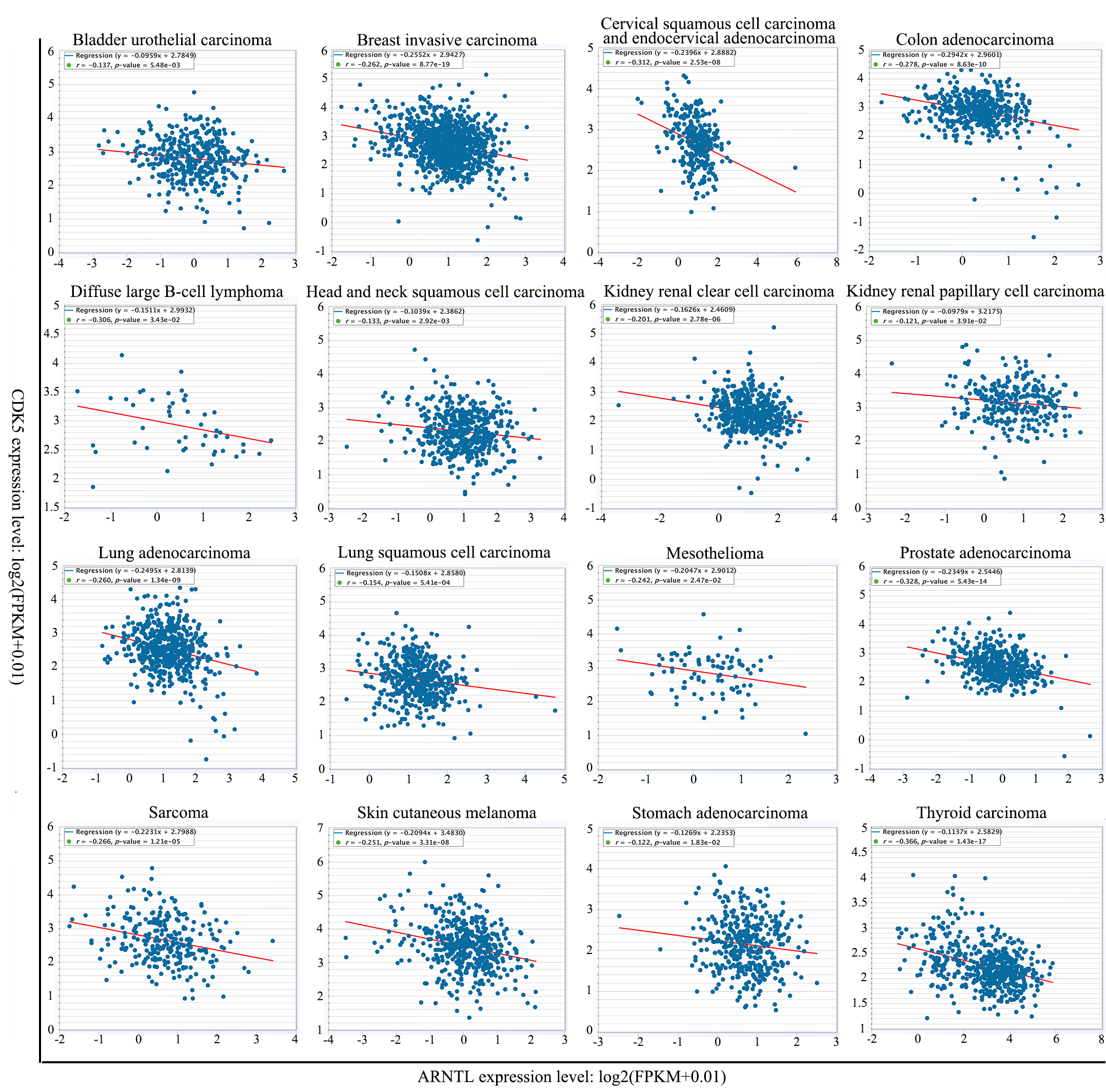

Supplement: Supplementary file 6 — Figure S5. Correlation between ARNTL expression and CDK5 expression in other cancers using The Cancer Genome Atlas (TCGA) dataset analysis. (TIF 1391 kb) [file 13046_2018_997_MOESM6_ESM.tif]

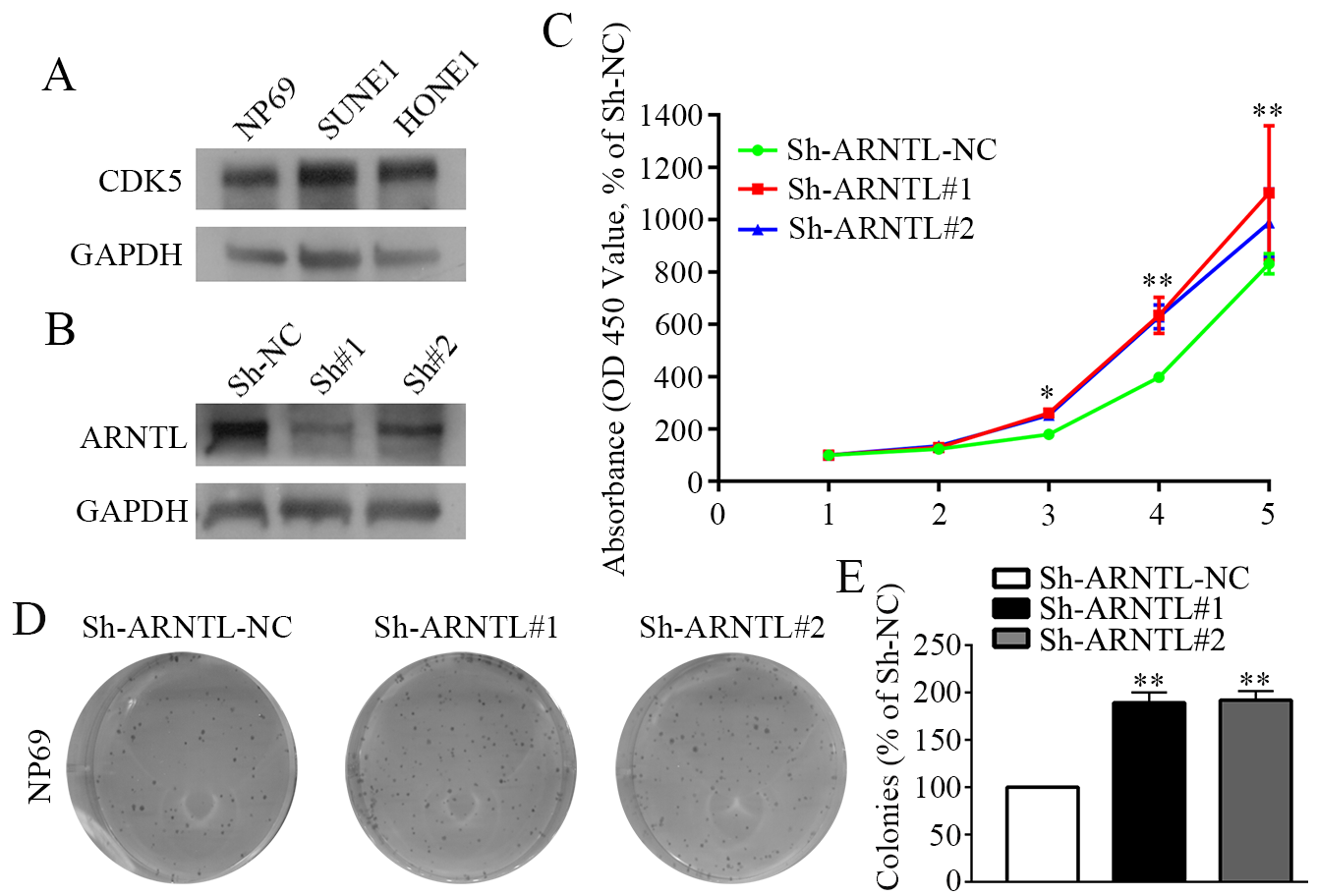

Supplement: Supplementary file 7 — Figure S6. ARNTL affect the proliferation ability of NP69 cells. (A) Western blot assay found that CDK5 is downregulated in NP69 cells; (B) Knocking down ARNTL expression in NP69 cells; (C) CCK-8 assay demonstrated that knocking down ARNTL could promote NP69 cells proliferation; (D-E) Colony formation assay showed that knocking down ARNTL could promote NP69 cells proliferation. (TIF 3612 kb) [file 13046_2018_997_MOESM7_ESM.tif]
