# Supplementary figures and images for: Correction to: ARNTL hypermethylation promotes tumorigenesis and inhibits cisplatin sensitivity by activating CDK5 transcription in nasopharyngeal carcinoma
Source: J Exp Clin Cancer Res. 2022 Jan 21;41:30. doi: 10.1186/s13046-021-02238-5 (PMC8780747; doi:10.1186/s13046-021-02238-5)

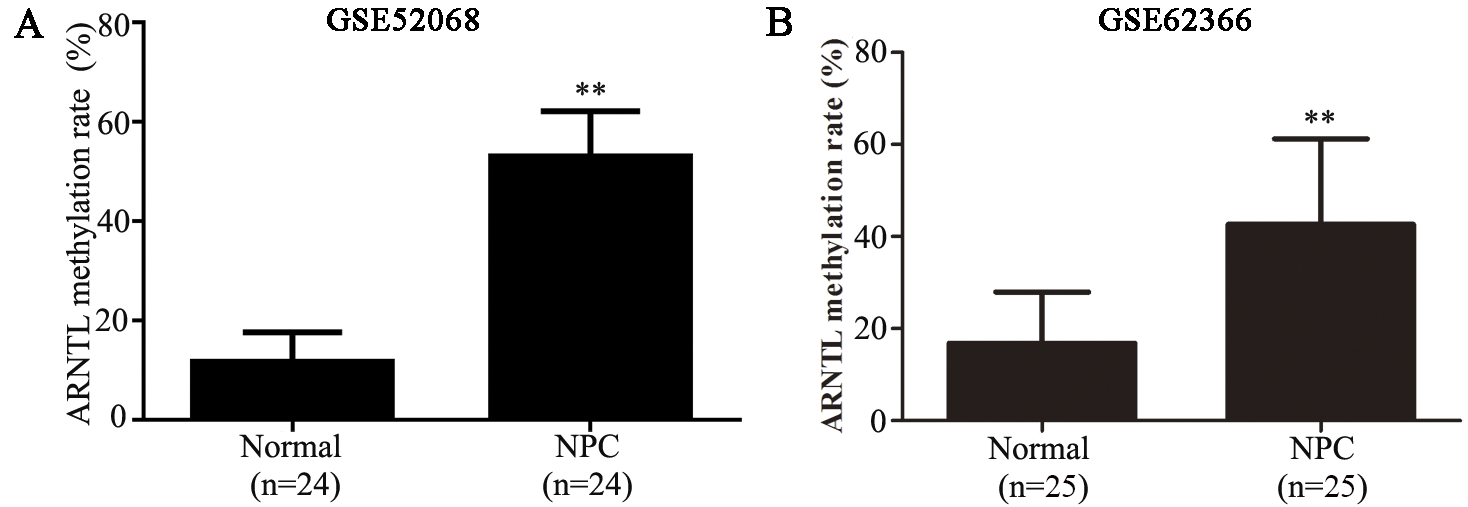

Supplement: Supplementary file 1 — Additional file 2: Fig. S1. ARNTL methylation levels in the GSE52068 and GSE62366 nasopharyngeal carcinoma datasets. [file 13046_2021_2238_MOESM2_ESM.tif]

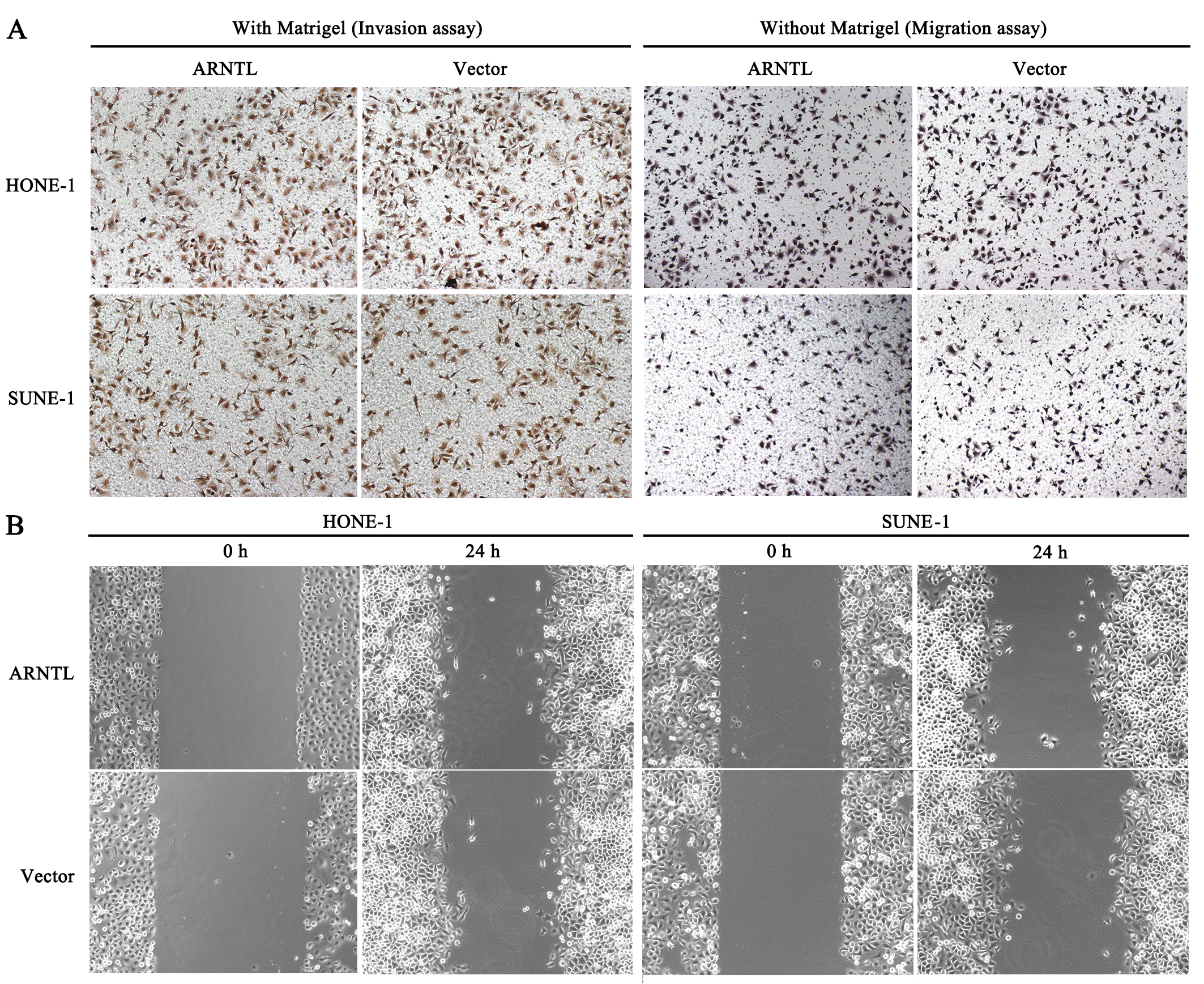

Supplement: Supplementary file 2 — Additional file 5: Fig. S4. Overexpression of ARNTL had no impact on nasopharyngeal carcinoma cells invasion and migration. (A) Images of Transwell invasion (left) and migration (right) assay with ARNTL-overexpression or Vector-overexpression SUNE1 and HONE1 cells. (B) Images of wound healing assay with ARNTL-overexpression or Vector-overexpression SUNE1 and HONE1 cells. [file 13046_2021_2238_MOESM5_ESM.tif]
